# SEM-Based Methods to Form Confidence Intervals for Indirect Effect: Still Applicable Given Nonnormality, Under Certain Conditions

**DOI:** 10.3389/fpsyg.2020.571928

**Published:** 2020-12-18

**Authors:** Ivan Jacob Agaloos Pesigan, Shu Fai Cheung

**Affiliations:** Department of Psychology, Faculty of Social Sciences, University of Macau, Taipa, China

**Keywords:** mediation, nonnormal, confidence interval, structural equation modeling, bootstrapping

## Abstract

A SEM-based approach using likelihood-based confidence interval (LBCI) has been proposed to form confidence intervals for unstandardized and standardized indirect effect in mediation models. However, when used with the maximum likelihood estimation, this approach requires that the variables are multivariate normally distributed. This can affect the LBCIs of unstandardized and standardized effect differently. In the present study, the robustness of this approach when the predictor is not normally distributed but the error terms are conditionally normal, which does not violate the distributional assumption of ordinary least squares (OLS) estimation, is compared to four other approaches: nonparametric bootstrapping, two variants of LBCI, LBCI assuming the predictor is fixed (LBCI-Fixed-X) and LBCI based on ADF estimation (LBCI-ADF), and Monte Carlo. A simulation study was conducted using a simple mediation model and a serial mediation model, manipulating the distribution of the predictor. The Monte Carlo method performed worst among the methods. LBCI and LBCI-Fixed-X had suboptimal performance when the distributions had high kurtosis and the population indirect effects were medium to large. In some conditions, the problem was severe even when the sample size was large. LBCI-ADF and nonparametric bootstrapping had coverage probabilities close to the nominal value in nearly all conditions, although the coverage probabilities were still suboptimal for the serial mediation model when the sample size was small with respect to the model. Implications of these findings in the context of this special case of nonnormal data were discussed.

Mediation model is now a popular kind of theoretical model in research. According to Google Scholar, about 19,500 entries from 2015 to 2020 had the keywords *mediation*, *mediating*, or *indirect effect* (as of May 8, 2020) even if the search was restricted to only entries with one of these terms in the titles. Measuring the indirect effect, the effect of a variable (the predictor) on another one (the outcome) through one or more mediators is useful because it can help us to assess the importance of different casual mechanisms, to test whether a posited mechanism is supported by data, and to obtain the interval estimation of the indirect effect using confidence intervals. For a simple mediation model with one predictor, one mediator, one outcome variable, and some control variables, the unstandardized indirect effect is the product of the unstandardized regression coefficient of the path from the predictor to the mediator (the *a* path) and that of the path from the mediator to the outcome variable (the *b* path). The standardized indirect effect (also called completely standardized indirect effect in Preacher and Kelley, 2011) is the product of the standardized coefficients of the *a* path and the *b* path. Preacher and Kelley argued that the preferred metric to use is the “metric that most effectively communicates the particular effect size in the specific context” (p. 95). However, the sample estimates of both unstandardized and standardized indirect effects are the products of two or more sample estimates of path parameters, resulting in a nonnormal sampling distribution with no simple analytic form (Craig, 1936). This makes forming the confidence interval for the indirect effects difficult. Various methods have been proposed to form an optimal confidence interval. In the present paper, for reasons to be presented later, we focus on two group of methods, namely, the regression-based nonparametric bootstrapping and the structural equation modeling (SEM)-based likelihood approach. We compare the performance of these methods in a special case of nonnormal data: nonnormal predictors. In this paper, we first present the simple mediation model and its estimation. We then discuss how the case of nonnormal predictors is different from other cases of nonnormal predictors. Next, we briefly present the methods to be examined and how nonnormal predictors may impact their performance. The simulation study conducted is described and the results are reported. Last, we discuss the implications of the findings on forming interval estimates of indirect effects.

## Simple Mediation Model and the Estimation of Indirect Effects

**FIGURE 1:**
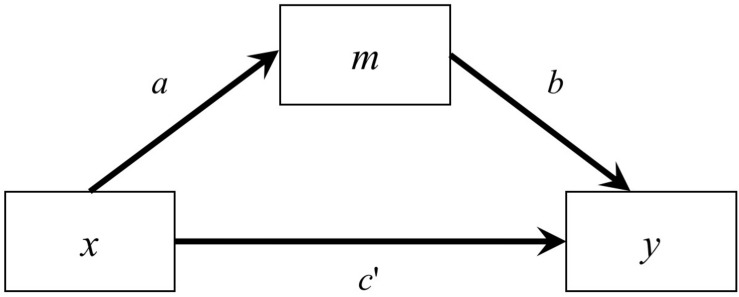
A Simple Mediation Model.

As shown in Figure 1, the classical simple mediation model has three variables, predictor (*x*), mediator (*m*), and outcome variable (*y*)^1^, and can be described by two regression models (Baron and Kenny, 1986; MacKinnon, 2008):

(1) $$m=i_{m}+a\,x+e_{m},$$

(2) $$y=i_{y}+b\,m+c^{\prime }\,x+e_{y}$$

where *e*_*m*_ and *e*_*y*_ are the error terms, *i*_*m*_ and *i*_*y*_ are the intercepts, and *a*, *b*, and *c*′ are the unstandardized regression coefficients. The outcome variable can also be expressed in terms of the predictor and the two error terms only:

$$y=i_{y}+b\,\left(i_{m}+a\,x+e_{m}\right)+c^{\prime }\,x+e_{y}$$

(3) $$=\left(i_{y}+b\,i_{m}\right)+\left(a\,b+c^{\prime }\right)\,x+\left(b\,e_{m}+e_{y}\right)$$

If we standardize all variables in Eqs. 1 and 2, the corresponding standardized models are (see also MacKinnon, 2008, and Preacher and Kelley, 2011):

(4) $$z_{m}=a_{z}\,z_{x}+e_{z_{m}},$$

(5) $$z_{y}=b_{z}\,z_{m}+c_{z}^{\prime }\,z_{x}+e_{z_{y}},$$

where *e*_*z_m*_ and *e*_*z_y*_ are the error terms, and *a*_*z*_, *b*_*z*_, and $c_{z}^{\prime }$ are the standardized regression coefficients.

The unstandardized indirect effect is equal to *ab*. The standardized indirect effect is then *a*_*z*_*b*_*z*_. The standardized indirect effect can also be computed by the following equation (called completely standardized indirect effect in Preacher and Kelley, 2011):

(6) $$a_{z}\,b_{z}=\frac{\sigma_{x}}{\sigma_{y}}\,a\,b,$$

where σ*_*x*_* and σ*_*y*_* are the standard deviations of *x* and *y*, respectively.

Interval estimation of the indirect effects is more complicated than point estimation. When the parameters in the two models are estimated from the sample data, there are well-established methods to estimate the parameters, such as using ordinary least squares (OLS) on the two regression models, or using maximum likelihood to fit the model as one single path model. However, the sampling distribution of the sample *ab* is nonnormal and does not have a simple analytic form (Craig, 1936). The standardized indirect effect has one complication on top of the complications in relation to the unstandardized indirect effect: the standardizers (the standard deviations of the predictor and the outcome) are used in the computation but they themselves are sample statistics. The sampling distribution of the standardized indirect effect is even more complicated than that of the unstandardized indirect effect, as suggested by the results by Yuan and Chan (2011) on the sampling distribution of standardized regression coefficients in a multiple regression model.

Various methods to form the confidence interval of indirect effects have been proposed and empirically studied (see Hayes and Scharkow, 2013 for a review). In the comprehensive studies by Cheung (2009a, b), some methods were found to have unsatisfactory performance in forming the confidence intervals of a standardized indirect effect, even for normally distributed variables and residuals. Therefore, we only selected methods that have been shown to perform satisfactorily for standardized indirect effect and examined their performance when the predictor was not normally distributed.

Currently, it seems that the most popular method is nonparametric bootstrapping (Bollen and Stine, 1990; Shrout and Bolger, 2002; Preacher and Hayes, 2004). This method, to be elaborated below, involves resampling the raw data and forming an empirical distribution of the indirect effect point estimates to form the confidence interval of an indirect effect. We selected this method for examination because it has been found to perform well across various situations and for both standardized and unstandardized indirect effects (e.g., MacKinnon et al., 2004; Cheung, 2009a, b; Biesanz et al., 2010) and can serve as a benchmark to assess the performance of the other approaches, presented next.

Another approach selected is the likelihood-based confidence interval (LBCI) in SEM (Neale and Miller, 1997; Cheung, 2009a, b; Falk and Biesanz, 2014), to be elaborated after the discussion of nonnormal predictors. Like nonparametric bootstrapping, this method does not require the knowledge of the sampling distribution of the indirect effect. Unlike nonparametric bootstrapping, this method is parametric and relies on the distributional assumptions made by an estimation method. Although not as popular as the nonparametric bootstrapping, this method has its own advantages. It does not require the raw data and does not involve resampling.

The last approach selected was the Monte Carlo method (Preacher and Selig, 2012). Like the LBCI approach, this method does not require the raw data and does not involve resampling. It only needs to estimate standard errors and the point estimates. It is even simpler than the LBCI approach because it does not involve re-estimating the model. Although less popular than nonparametric bootstrapping, a crude search in Google Scholar for papers cited Preacher and Selig (2012) and had the keyword *Monte Carlo* returned over 600 entries on or after 2016. Preacher and Selig found that this method performed as good as nonparametric bootstrapping for unstandardized indirect effects. However, this method was not included in the comprehensive simulation studies by Cheung (2009a, b). Therefore, we also selected this method for investigation in the present study.

## Nonnormal Predictors

Although many statistical techniques assume that the variables of concern are normally distributed or multivariately normally distributed, there are also many variables that are not normally distributed. Although we labeled all these distributions that deviate from the normal distribution (univariate or multivariate) as *nonnormal* distributions, we would like to remark that this group of distributions encompasses a wide variety of distributions. Some are symmetric but have heavy tails (e.g., *t* distribution), some are asymmetric (e.g., the distribution of monthly salary), and some are uniform (e.g., the distribution of students across different years of studies). Therefore, in the following sections, we are not assuming that all nonnormal distributions are the same, as will become apparent when we presented the types of nonnormal distributions we investigated.

Despite the name “nonormal,” nonnormal data are more popular than usually believed. In the seminal paper by Micceri (1989), an investigation of over 400 samples in psychology found that nonnormality, rather normality, was the norm. A recent study by Blanca et al. (2013) of nearly 700 distributions also led to the same conclusions. There are various possible reasons for nonnormality. In some cases, the nature of a variable results in nonnormality (e.g., the scores on a clinical state such as depression or gambling problems, or individual monthly salary). In some other cases, nonnormality may be the result of data collection methods, even of unbiased ones (e.g., recruiting similar numbers of students from each year of study in a university, or similar numbers of participants across different age groups).

The impact of nonnormal variables on forming confidence interval of indirect effects have been studied in various situations (e.g., Biesanz et al., 2010; Falk, 2018). However, most of them studied the case in which the distributional assumption of an estimation method is violated. For example, Biesanz et al. (2010) studied the case of nonnormal errors, which violates the assumption of normal errors for OLS estimation and so even the standard errors of the regression coefficients used to compute the product are incorrect. Falk (2018) studied the case of latent mediation model using SEM, and all the variables are nonnormal. However, there is a scenario, to be discussed next, that has all variables not multivariate normal but the distributional assumption of OLS estimation and maximum likelihood estimation are actually not violated.

As discussed above, variable can be nonnormal by nature, or nonnormal because of the research design. Let us consider the case in which the distribution of both error terms in the two regression models in the simple mediation model are normally distributed, or at least not too nonnormal and the assumption of normal distribution is a good approximation. Even though nonnormal variable is common, in some situations, it is theoretically reasonable to expect that the *error* is approximately normally distributed, if ceiling effect and floor effect are absent and the error is a consequence of many unmeasured factors combined. The predictor, however, can be nonnormal for reasons described above. It also differs from the error because it represents one attribute or a group of homogeneous attributes, instead of an aggregation of many factors. If the predictor is nonnormal, then the distribution of the mediator conditioned on the predictor is still normal, but the marginal distribution of the mediator is nonnormal. Similarly, the distribution of the outcome variable conditioned on the predictor is normal, but the marginal distribution of the outcome variable is nonnormal. Consequently, the joint distribution of the three variables are multivariate nonnormal.

**FIGURE 2:**
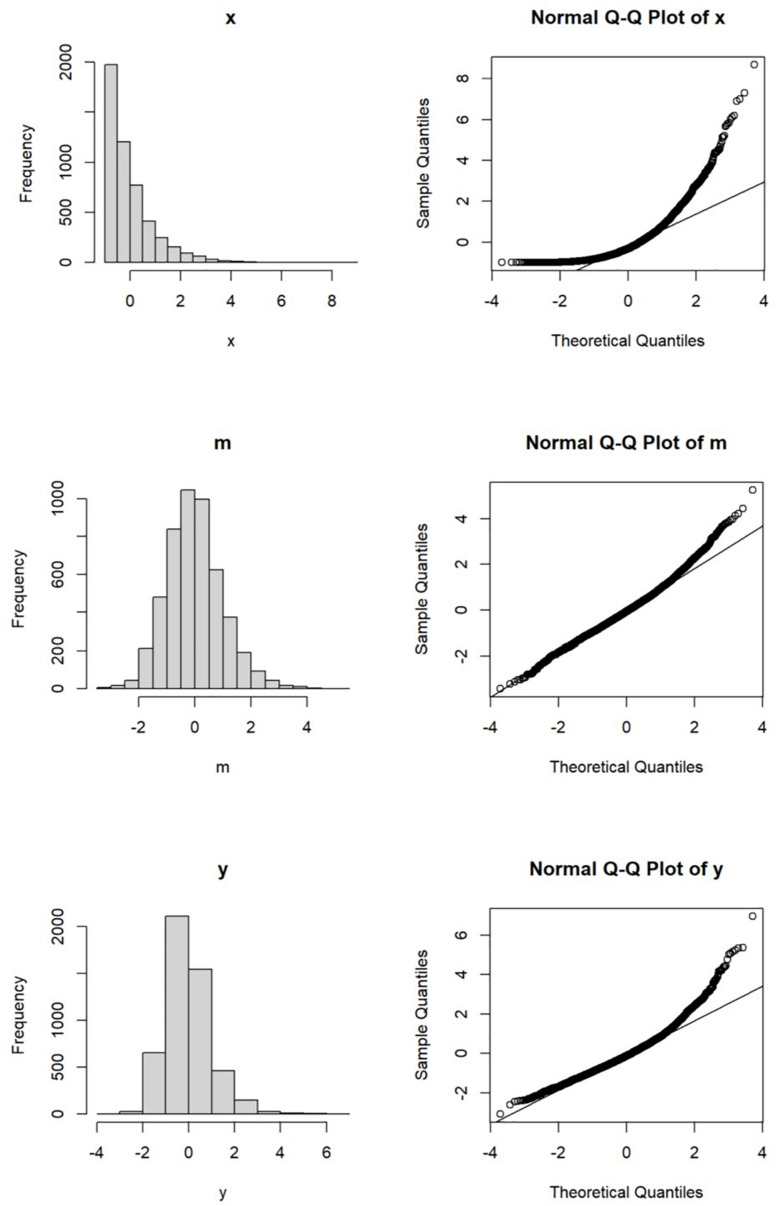
Distributions of *x*, *m* and *y.*

For illustration, let us consider a sample of 5000 cases (data generation R script available as supplementary Material). The predictor is exponentially distributed and rescaled to have zero population mean and population standard deviation equal to one. The univariate distribution and the normal Q–Q plot are shown in Figure 2.

Suppose the predictor has an effect of 0.60 on the mediator and the error is normally distributed with variance set to 1 −0.60^2^ or 0.64 such that its population variance is one. Its univariate distribution and normal Q–Q plot are shown in Figure 2. Though not as skewed as the predictor, the distribution of the mediator is slightly nonnormal, with skewness equal to 0.45 and excess kurtosis equal to 0.81.

Suppose the outcome variable is influenced by both the mediator and the predictor, with the effects from the mediator and the predictor equal to 0.60 and 0.40, respectively. Again, the error of the outcome variable is normally distributed, with variance set to 0.192 such that the population variance of the outcome variable is one. The univariate distribution and the normal Q–Q plot of the outcome variable are presented in Figure 2.

Compared to the mediator, the distribution of the outcome variable is more nonnormal, partly because of the direct effect from the nonnormal predictor. The skewness of the outcome variable is 0.95 and its excess kurtosis is 2.33, both larger than those of the mediator. It can be inferred that the joint multivariate distribution of these three variables is nonnormal. However, as discussed below, this does not necessarily violate the distributional assumptions of OLS estimation and maximum likelihood estimation.

**FIGURE 3:**
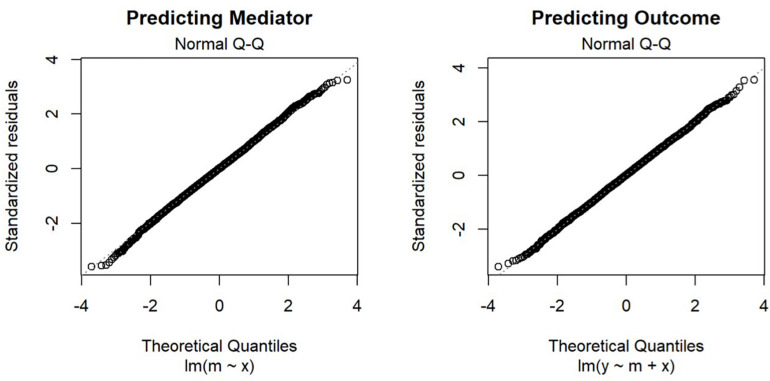
Normal Q-Q Plot of Residuals.

First, in OLS estimation, the distributional assumption is on the conditional distribution of the error (Fox, 2016), not on the predictors. The estimates of the standard errors of the unstandardized regression coefficients are consistent even if the predictors are not normally distributed (Yuan and Chan, 2011). This is obvious when we consider the well-established practice of using dummy variables in linear regression, in which the dummy variables certainly cannot be normally distributed, and the joint distribution of the predictors and the outcome variables cannot be multivariate normal. As shown in Figure 3, if we examine the normal Q–Q plots of the residuals of the two regression models, their distributions are close to normal, as we already know from the way we generate the error.

Second, this case also does not violate the distributional assumption for maximum likelihood estimation in the SEM-based approach. Although it is usually believed that maximum likelihood estimation assumes the variables to have a multivariate normal distribution, in path analysis models, if (a) the exogenous variables and the errors are independent, (b) the joint distribution of the endogenous variables (precisely speaking, the error terms of these variables in the structural models of observed variables) conditional on the exogenous variables (the predictors in a path model) are multivariate normal, and (c) the distribution of the exogenous variables are not functions of model parameters (other than their own variances and covariances), minimizing the usual maximum likelihood discrepancy function still leads to the maximum likelihood estimators of the parameters involving the endogenous variables, such as the path coefficients from the exogenous variables to the endogenous variables and the error variances and covariances of the endogenous variables (see Bollen, 1989, pp. 126–127; also see Jöreskog, 1973, pp. 94–95). In other words, when a path analysis model, such as the simple mediation model, is fitted and estimated by maximum likelihood, the required distributional assumption for maximum likelihood estimation of the path coefficients is similar to that for OLS estimation of one single regression model, although more restrictive because it is on the joint distribution of the error terms.

To our knowledge, this kind of nonnormality is rarely investigated. In the next section, we briefly present the two approaches to form the confidence interval for indirect effects, and how this kind of nonnormality may or may not affect their performance. In view of how this kind of nonnormality may affect the LBCIs for unstandardized and standardized indirect effects differentially, we also present two variants of LBCI, as well as the Monte Carlo method.

### Robustness to Nonnormality

Despite our discussion above on nonnormality, we would like to remark that, based on central limit theorem, some parametric methods that assume normally distributed error are still valid even if this assumption is violated, when the sample size is large enough. For example, in the simple case of a sample mean, it is well known that, as the sample size increases, the sampling distribution of the sample mean approaches normality even when the population distribution is not normal. In the case of OLS estimation in multiple regression, the significance tests and confidence intervals are approximately correct when the sample size is large enough, even when the assumption of normality for the residuals is violated (Fox, 2016). In the present study, rather than examining cases in which the assumption of normality is violated, we examined cases in which this assumption is not violated in estimating the model parameters, but may be violated when estimating *derived parameters* such as the standardized indirect effects.

## Procedures Examined

### Nonparametric Bootstrapping

Bootstrapping method (Efron and Tibshirani, 1993) has been used widely in forming the confidence interval for a sample indirect effect. The most popular implementation in mediation analysis is perhaps nonparametric bootstrapping, partly because it can be implemented easily by the tool developed by Hayes (2017). For a sample of *N* cases, a bootstrap sample is generated by randomly drawing *N* cases from this sample with replacement. The indirect effect is then estimated from this bootstrap sample. These steps are repeated *K* times to generate *K* bootstrap estimates of the indirect effect. Various methods have been proposed to form the confidence intervals. Percentile method is the simplest one, using the 2.5th and 97.5th percentiles to form the 95% confidence interval. This is also the current default method used in the SPSS PROCESS macro by Hayes (2017). Interested readers are referred to other references for more information on nonparametric bootstrapping (e.g., Efron and Tibshirani, 1993; Efron and Hastie, 2017).

Previous studies found that this simple method performed as well as more complicated methods, if not better (e.g., MacKinnon et al., 2002; Fritz et al., 2012). Bootstrapping is attractive because it is simple to implement. It has been shown to perform well in the interval estimation of both unstandardized and standardized indirect effects (MacKinnon et al., 2004; Cheung, 2009a). This method does not assume the variables in the model to have any particular joint distribution (Efron and Tibshirani, 1993). When the predictor is not normally distributed in the population, this method should also have optimal performance for both unstandardized and standardized indirect effects. Therefore, we selected this method as the benchmark for evaluating the performance of the other methods.

### Likelihood-Based Confidence Interval

Another method to form the confidence interval for the indirect effect adopts a SEM approach and uses the likelihood-based method (Cheung, 2007, 2009a; Falk, 2018). This is a general method for forming the confidence interval of an estimate using likelihood ratio test. Adopting Falk’s example (2018, see also Neale and Miller, 1997), suppose we want to form the 95% LBCI of the path coefficient θ in a model. Let the estimate be $\hat{\theta }$ when θ is freely estimated. We find two values, θ_*L*_ and θ_*H*_, the former higher than $\hat{\theta }$ and the latter lower than $\hat{\theta }$, such that the likelihood ratio tests between two alternative models, each with θ equal to one of these values, and the model with θ freely estimated have *p* value equal to 1 −0.95 or 0.05. The 95% LBCI is then θ_*L*_ to θ_*H*_. A model with θ equal to a value outside this range, such as lower than θ_*L*_ or higher than θ_*H*_, will be significantly different from the model with θ equal to its maximum likelihood estimate by the likelihood ratio test, with *p* < 0.05. The idea behind this method is forming the confidence interval by “inverting a test statistic” (Casella and Berger, 2001, p. 420). This method can be generalized to forming the confidence interval of a function of parameters (Pek and Wu, 2015), such as the unstandardized indirect effect, which is a function of *a* and *b*. Interested readers are referred to Casella and Berger (2001) for the theoretical justification, Pek and Wu (2015) on the technical details in implementation, and Falk and Biesanz (2014) on the algorithm to find the LBCI for a function of parameters, such as the indirect effect. In the present study, we used the OpenMx R package (Neale et al., 2015), which adopts the algorithm developed by Neale and Miller (1997) and Wu and Neale (2012).

When applied to form the LBCI of an unstandardized indirect effect, a mediation model is fitted as a structural equation model. A LBCI is then formed for the product of two parameters, the regression coefficients of the *a* path and the *b* path (Cheung, 2007).

(7) $${\mathrm{ab}}_{u\,n\,s\,t\,a\,n\,d\,a\,r\,d\,i\,z\,e\,d}=a\,b.$$

This method, theoretically, yields confidence limits within the bound of the parameter space and allows for asymmetric limits for the unstandardized indirect effect, as it should be due to the asymmetric sampling distribution of the product. Previous studies have found that it performed satisfactorily in giving the interval estimates of the unstandardized indirect effect (e.g., Cheung, 2007).

To form the confidence interval for the standardized indirect effect, an early implementation requires the formation of a special model with latent variables and nonlinear constraints (e.g., Cheung, 2009a). Recently, some SEM software packages (e.g., OpenMx) allow users to specify a derived parameter directly and compute its LBCI. In the simple mediation model, the standardized indirect effect can be expressed as a function of the following model parameters:

(8) $$a\,b_{s\,t\,a\,n\,d\,a\,r\,d\,i\,z\,e\,d}=a\,b\,\sqrt{\frac{\sigma_{x}^{2}}{b^{2}\,\sigma_{e_{m}}^{2}+\sigma_{e_{y}}^{2}+\sigma_{x}^{2}\,{\left(a\,b+c^{\prime }\right)}^{2}}},$$

where $\sigma_{x}^{2}$ is the variance of the predictor and $\sigma_{e_{m}}^{2}$ and $\sigma_{e_{y}}^{2}$ are the variances of the error terms of the mediator and the outcome variable, respectively. Other parameters have been defined in the presentation of the simple mediation model. The sample estimate of the standardized indirect effect is computed from the sample estimates of these parameters. This parameter is a function of more parameters than the unstandardized indirect effect, which is a function of only two parameters. Simulation studies have shown that the coverage probability of this interval is close to the nominal level when the variables had a multivariate normal distribution and maximum likelihood estimation was used (e.g., Cheung, 2009a).

Few studies examined the performance of LBCI for indirect effect when the predictor is drawn from a nonnormal distribution and maximum likelihood is used for estimation. Falk (2018) compared various methods for forming the confidence interval of the indirect effect for a latent variable mediation model when the observed variables are nonnormal and found that nonparametric bootstrapping with percentile confidence interval was on average the best methods among those compared, including a robust version of LBCI. We first discussed the case for unstandardized indirect effect. On the one hand, it can be argued that LBCI will have suboptimal performance because maximum likelihood estimation assumes multivariate normality, as in previous studies on latent variable mediation model. On the other hand, if only the predictor is nonnormal, it can be argued that maximum likelihood estimation, and subsequently LBCI, will still perform optimally because the conditional joint distribution of the error terms is still multivariate normal. The unstandardized indirect effect is a function of only two parameters, both path coefficients. As long as the likelihood ratio test that involves changing these two parameters is valid, the LBCI should be valid. This is the case for nonnormal predictor coupled with normal errors, as discussed above. In the present study, the performance of LBCI for unstandardized indirect effect was examined empirically.

The case for standardized indirect effect is different. As shown above, the standardized indirect effect is a function of six parameters, and one of them is the variance of the predictor. Therefore, the likelihood ratio test used for forming LBCI will involve estimating these six parameters. Even though maximum likelihood estimation of the three path coefficients and the two error variances does not require the predictor to be normally distributed, the maximum likelihood estimation of the sample variance of the predictor obviously assumes that the predictor is normally distributed. Therefore, unlike the unstandardized indirect effect, it is reasonable to expect that the performance of the LBCI of standardized indirect effect will be adversely affected by normally distributed predictors. However, unlike previous studies on nonnormal variables, only one of the six parameters is affected. Therefore, practically, the impact may not be as large as in previous studies of nonnormal variables. In the present study, the degree of impact of nonnormality in predictors on the LBCI for the standardized indirect effect was examined empirically.

In short, it is not clear whether and how the LBCI for the unstandardized indirect effect will be affected by nonnormal predictors coupled with normal errors, while it is plausible that the LBCI for the standardized indirect effect will have suboptimal performance in this case. Two possible variants of LBCI that may be applicable when the predictor is not normally distributed will be presented below.

### LBCI With ADF (LBCI-ADF)

The likelihood-based method is not confined to maximum likelihood estimation. As long as likelihood ratio test can be done appropriately using a one-*df* chi-square test, LBCI can be formed (Pek and Wu, 2015). Therefore, if the data are nonnormal, other estimation methods can be used and the LBCI is formed in the same manner. For example, Pek and Wu suggested that the asymptotically distribution free (ADF) method (Browne, 1984) can be used for nonnormal data. As an initial investigation, we follow their suggestion and study the performance of LBCI based on ADF, which we call LBCI-ADF in the present paper. We selected this approach because it is readily available in OpenMx (Neale et al., 2015), one of the common R packages for forming LBCI, allowing researchers to use this method with no additional programming. ADF is rarely used in practice because the required sample size is large (Hu et al., 1992; Curran et al., 1996). However, the models we studied only have three to four variables and therefore the range of sample sizes we studied may be sufficient for ADF. If this method is promising, then further studies can examine other robust methods in forming the confidence interval of the indirect effect with nonnormal predictors.

### LBCI With Fixed X (LBCI-Fixed-X)

If one suspects that the predictor (*x*) is nonnormally distributed, one may consider fitting a path model, fixing the variance of the predictor to its observed value, and computing the LBCI for the unstandardized and standardized indirect effects. In other words, when searching for the confidence limits, only the other five parameters used to compute the standardized indirect effect will change. Setting exogenous observed variables as fixed variables is also the default option in some statistical functions (e.g., lavaan, Rosseel, 2012). This sounds like a viable option because the maximum likelihood estimator, as described above, can also be derived when all exogenous observed variables (only one in this case, the predictor) are treated as fixed and the conditional joint distribution of the error terms are multivariate normal (Bollen, 1989). In other words, nonnormality of the predictor should not affect the validity of the confidence interval.

However, if an exogenous variable is assumed to be fixed, then its variance (and standard deviation) is also assumed to be fixed. This may affect the interval estimation of the standardized indirect effect if the predictor is actually stochastic rather than fixed, as is the case in most studies. The sampling error of the variance of the predictor is not taken into account when forming the LBCI for the standardized indirect effect. Therefore, this method, while avoiding the problem of nonnormality, may suffer from the problem of fixed variance when the predictor is stochastic. The net impact of nonnormality on this method needs to be examined empirically.

### Monte Carlo

The last method is the Monte Carlo. Like nonparametric bootstrapping, it is a simulation-based method. However, this method does not need the raw data. This method only needs the point estimates and standard errors of the *a* path and the *b* path. If the assumptions of OLS estimation are met, the sampling distribution of these two parameters are normal. In the Monte Carlo method, a large number of pairs of random numbers from a bivariate normal distribution are generated, with the means and standard deviations of this distribution equal to the point estimates and standard errors of the two parameters. Previous studies (MacKinnon et al., 2004; Preacher and Selig, 2012) have set the covariance to zero following the assumption that, in the case of the simple mediation model, the covariance between *a* path and *b* path is zero (Sobel, 1982). The distribution of the product of these pairs of random numbers is then used to form the confidence interval of the indirect effect. For example, for a 95% confidence interval, the 2.5th and 97.5th percentiles of the distribution are the lower and upper confidence limits, respectively. The Monte Carlo method has been shown to have satisfactory performance in forming the confidence interval of the unstandardized indirect effect (MacKinnon et al., 2004; Preacher and Selig, 2012).

This method is based on the rationale that the two OLS estimates are normally distributed, which are the assumptions made implicitly when researchers interpret the confidence intervals of the *a* and *b* paths in OLS estimation. In OLS estimation, no distributional assumption is needed for the predictors. Therefore, even if the predictor is not normally distributed, if all other assumptions are met, the sample unstandardized regression coefficients are still normally distributed. It is expected that the Monte Carlo method will also perform satisfactorily in interval estimation of the unstandardized indirect effect with nonnormal predictors.

To our knowledge, this method has not yet been extended to standardized indirect effect. One problem is the standardizer. The standardized indirect effect is computed by multiplying the product by the standard deviation of the predictor and dividing it by the standard deviation of the outcome (Eq. 6). Even if the sampling distribution of the sample standard deviation of the predictor can be simulated by the Monte Carlo method, the sampling distribution of the sample standard deviation of the outcome cannot be easily simulated because it is a function of the standard deviations of the predictor and the error terms. One possible option is to assume that the standardizers are fixed. We suspect that this was how the Monte Carlo confidence interval for the standardized indirect effect was computed in some previous studies. Therefore, we also adopted this method for standardized indirect effects for completeness. However, this approach ignored the sampling variances and covariances of the sample standard deviations of the predictor and outcome. Therefore, this method may fail to work in forming the confidence interval for the standardized indirect effect. Its performance on the standardized indirect effect needs to be empirically investigated.

## Materials and Methods

### Models

**FIGURE 4:**
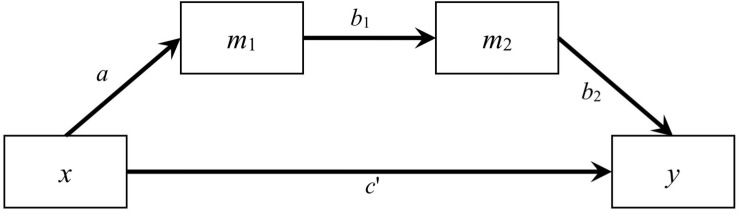
A Serial Mediation Model with Two Mediators.

In this study, we examined two models: a simple mediation model with one predictor, one mediator, and one outcome variable, and a serial mediation model with one predictor, two mediators, and one outcome variable. We restricted to just one predictor to avoid confounding from other factors such as control variables and inter-predictor correlations. For the simple mediation model, the path from the predictor to the mediator was denoted as *a*, the path from the mediator to the outcome variable was denoted as *b*, and the path from the predictor to the outcome variable was denoted as *c*′ (see Figure 1). For the serial mediation model (Figure 4), the path from the predictor (*x*) to the first mediator (*m*_1_) was denoted as *a*, the path from the first mediator to the second mediator (*m*_2_) was denoted as *b*_1_, the path from the second mediator to the outcome variable (*y*) was denoted as *b*_2_, and the direct path from the predictor to the outcome variable was denoted as *c*′. The population values of all other paths were zero, although they were still estimated in the simulation, such that the model being fitted were saturated in both conditions. The population distribution of the predictor was manipulated, while the errors of the mediators and the outcome were generated from normal distributions. The serial mediation model had one more variable, resulting in four more elements in the variance–covariance matrix and four more parameters, allowing us to examine the impact of number of variables on the estimation methods, especially the LBCI-ADF method, which was known to require a large sample size.

### Factors Examined

#### Sample Size (*n*)

The same five levels of sample size examined in Cheung (2009a) were examined: 50, 100, 150, 200, and 500. This range should cover the sample sizes found in most studies of mediation effects and also ensure that the results in the present study could be compared directly with those in Cheung (2009a).

### Indirect Effect (*ab* and *ab*_1_*b*_2_)

Cheung (2009a) adopted the rule of thumb proposed by Cohen (1988, pp. 413–414) for small, medium, and large effects for *R*^2^ (0.02, 0.13, and 0.26) and derived that the corresponding standardized indirect effects (*ab*s) are 0.141, 0.361, and 0.510. If *a* and *b* paths are equal, the corresponding levels of *a* and *b* paths are 0.376, 0.600, and 0.714. The condition of nil indirect effect (*a* = *b* = 0) was also examined. For the simple mediation model, we extended the conditions to unequal *a* and *b*, by setting one of the paths to the largest level examined (0.714), resulting in these four pairs of values: *a* = 0.1975 and *b* = 0.714, *a* = 0.714 and *b* = 0.1975, *a* = 0.5056 and *b* = 0.714, as well as *a* = 0.714 and *b* = 0.5056. The standardized indirect effects of the first two pairs are 0.141 (small effect), and the standardized indirect effects of the last two pairs are 0.361 (medium effect). The total number of combinations of *a* and *b* values is eight. For the serial mediation model, we constrained *b*_1_ and *b*_2_ to be equal in all conditions. Conditions for the serial mediation model shared the same combinations of levels as in the simple mediation model, with the square root of the population value *b* used as the population values of *b*_1_ and *b*_2_. Therefore, the levels of indirect effect examined were the same for both models, even though they differ on the number of mediators.

#### Direct Effect (*c*′)

The levels of direct effect (*c*′) were chosen such that the population *R*^2^ increases when adding the predictor to the regression model after the mediation has been included (that is, adding the *c*′ path) were 0.00, 0.02, 0.13, and 0.26, following the rule of thumbs for the *R*^2^ above. Given the *R*^2^ increase (*d*), *c*′ depends only on *d* and *a*: *c*′ = *d/*(1 − *a*^2^). For some combinations of *a*, *b*, and *c*′, the total *R*^2^ values were unusually high. Therefore, we dropped combinations that result in an implied population *R*^2^ larger than 0.80 (corresponding to a multiple correlation of 0.89).

#### Distributions of Predictors

Five distributions of the predictor were examined: normal distribution (skewness = 0, excess kurtosis^2^ = 0), exponential distribution (rate = 1, skewness = 2, excess kurtosis = 6), beta distribution (α = β = 1.5, skewness = 0, excess kurtosis = −1), *t* distribution with *df* = 5 (skewness = 0, excess kurtosis = 6), and *t* distribution with *df* = 6 (skewness = 0, excess kurtosis = 3). These distributions include five kinds of situations: normal distribution, positively skewed distribution, symmetric distribution with light tails, symmetric distribution with heavy tails, and symmetric distribution with moderately heavy tails. We selected these specific nonormal distributions because they reflect situations that researchers may encounter in their contexts. For example, response times often follow an exponential distribution; variables that are bell-shaped and symmetric might have extreme values resulting in positive excess kurtosis, which can be more appropriately modeled with a *t* distribution, using the *df* to control the degree of excess kurtosis; and lastly, scale scores computed from the means of item responses that have lower and upper limits can be more appropriately modeled with a beta distribution with α = β = 1.5. The generated random values for the predictor were rescaled such that the population means and standard deviations of all four distributions are identical (see below).

### Data Generation

The R software environment for statistical computing (R Core Team, 2018) was used to generate the raw data. For each condition, the required number of raw predictor scores (*x*) were generated using the corresponding R function (rnorm for normal distribution, rexp for exponential distribution, rbeta for beta distribution, and rt for *t* distribution). The scores were rescaled using the distribution’s population mean and standard deviation such that the population mean and standard deviation after rescaling were zero and one for all four distributions. For example, for the simple medication model (implemented by gendata in the package used for this study), raw mediator scores (*m*) were then generated by *m* = *ax*+ *e*_*m*_, where *e*_*m*_ scores were generated from a normal distribution with population mean equal to zero and population standard deviation equal to $\sqrt{1-a^{2}}$. This ensures that the population mean and standard deviation of *m* were also zero and one, respectively. Last, the outcome scores (*y*) were generated by *y* = *bm* + *c*′*x* + *e*_*y*_, where *e*_*y*_ scores were generated from a normal distribution with population mean equal to zero and population standard deviation equal to $\sqrt{1-b^{2}-c^{2}-2\,b\,c\,a}$. This ensures that the population mean and standard deviation of *y* were also zero and one, respectively. In sum, for all four distributions, the population means and standard deviations of *x*, *m*, and *y* are all equal to zero and one, respectively. The computation was slightly more complicated in the serial mediation. Interested readers can refer to the function gendata_serial in the package used for the present study for the details.

To simulate real-life situation with nonzero population means and standard deviations not equal to one, for the simple mediation model, the three variables were then rescaled to have population means equal to three, five, and four for *x*, *m*, and *y*, respectively, and population standard deviations equal to four, five, and three for *x*, *m*, and *y*, respectively. For the serial mediation model, population means were five and two for *m*_1_ and *m*_2_, respectively, and population standard deviations were five and two for *m*_1_ and *m*_2_, respectively. These values are arbitrary and would not affect the results, but would make the unstandardized coefficients and standardized coefficients different, serving as a safe guard against programming errors that would result in treating one as the other.

The package used to generate the data and do the analysis, as well the summary data, can be found at the OSF page for this manuscript^3^.

### Implementing the Selected Methods

#### Nonparametric Bootstrapping

R functions were used to implement nonparametric bootstrapping. For each replication, 2000 bootstrap samples were generated. For the simple mediation model, in each bootstrap sample, OLS linear regression was used to estimate the unstandardized and standardized *a* and *b* by fitting two models: regressing the mediator on the predictor, and regressing the outcome on both the mediator and the predictor. For the serial mediation model, three models were fitted: regressing the first mediator on the predictor, regressing the second mediator on the first mediator and the predictor, and regressing the outcome on all mediators and the predictors. The unstandardized *a*, *b*_1_, and *b*_2_ are estimated from these three models. The unstandardized and standardized indirect effects were computed for each bootstrap sample. The 2.5th and 95th percentiles of these two estimates in the 2000 bootstrap samples were used to form the confidence intervals for this replication. This method was implemented by the function boot_ci and boot_ci_serial in the package used in the present study.

#### LBCI

In each replication, OpenMx was used to fit a saturated partial mediation model using maximum likelihood estimation. Two user parameters were specified in the model specification using mxAlgebra. For the simple mediation model, the unstandardized indirect effect was simply the product of *a* and *b*, given by Eq. 7, and the standardized indirect effect was given by Eq. 8. The denominator in the term inside the square root is the variance of the outcome variable (see Appendix A of Cheung, 2009a). For the serial mediation, the unstandardized indirect effect was simply the product of *a*, *b*_1_, and *b*_2_. The standardized indirect effect was given by:

(9) $$a\,b_{1}\,b_{2_{s\,\tan\,d\,a\,r\,d\,i\,z\,e\,d}}=a\,b_{1}\,b_{2}\,\sqrt{\frac{\sigma_{x}^{2}}{\sigma_{e_{m_{1}}}^{2}\,{\left(b_{1}\,b_{2}\right)}^{2}+\sigma_{e_{m_{2}}}^{2}\,b_{2}^{2}+\sigma_{e_{y}}^{2}+\sigma_{x}^{2}\,{\left(a\,b_{1}\,b_{2}+c^{\prime }\right)}^{2}}}$$

where $\sigma_{x}^{2}$ is the variance of the predictor and $\sigma_{e_{m_{1}}}^{2}$, $\sigma_{e_{m_{2}}}^{2}$, and $\sigma_{e_{y}}^{2}$ are the variances of the error terms of the first mediator, the second mediator, and the outcome, respectively. The denominator in the term inside the square root is the variance of the outcome implied by the serial mediation model. The 95% likelihood-based confidence intervals for the unstandardized and standardized indirect effects were computed by mxCI in OpenMx (Neale and Miller, 1997; Wu and Neale, 2012). This method was implemented by the functions lb_ci_observed_free and lb_ci_observed_free_serial in the package used in the present study.

#### LBCI-ADF

This method was implemented as in the LBCI method above, except that the asymptotic distribution free method (called weighted-least squares in OpenMx) was used as the estimation method, using mxFitFunctionWLS (weights = “WLS”) to set the fit function (Neale et al., 2015). The 95% likelihood-based confidence intervals of the unstandardized and standardized indirect effects were computed as in LBCI above. This method was implemented by the functions lb_ci_wls and lb_ci_wls_serial in the packages used in the present study.

#### LBCI-Fixed-X

For each replication, a partial mediation model was fitted as in the LBCI method above, except that the variance of the predictor $\left(\sigma_{x}^{2}\right)$ was fixed to the sample variance of the predictor in each replication. The 95% likelihood-based confidence intervals of the unstandardized and standardized indirect effects were computed as in LBCI above. This method was implemented by the functions lb_ci_observed and lb_ci_observed_serial in the package used in the present study.

#### Monte Carlo

For each replication, OLS regression was used as in nonparametric bootstrapping to estimate the unstandardized *a* and *b* (*a*, *b*_1_, and *b*_2_ for the serial mediation model) and their standard errors. We then generated 1000 sets of random numbers from two normal distributions (three for the serial mediation model), using the sample estimates of unstandardized *a* and *b* (*a*, *b*_1_, and *b*_2_ for the serial mediation model) as the means and their standard errors as the standard deviations, as in Preacher and Selig (2012), Eq. 9. For the simple mediation model, for each set of Monte Carlo *a* and *b*, the product *ab* was computed. For the serial mediation model, the product *ab*_1_*b*_2_ was computed. The 2.5th and 97.5th percentiles of these 1000 products were then used to form the confidence interval for the unstandardized indirect effect. To form the Monte Carlo confidence interval for the standardized indirect effect, the confidence limits were multiplied by the sample standard deviation of the predictor and divided by the sample standard deviation of the outcome. For the technical details, please refer to the functions mc_ci (for simple mediation model) and mc_ci_serial (for the serial mediation model) in the package used in the present study.

### Assessment of Performance

For each method in a condition, the coverage probability, defined as the proportion that a 95% confidence interval includes the true population value, was computed. The nominal coverage probability for a 95% confidence interval is 0.95. We used the interval 0.935 to 0.965 (Serlin and Lapsley, 1985; Serlin, 2000; Biesanz et al., 2010; Falk and Biesanz, 2014) as a criterion for robustness to assess how close the coverage probability is to the nominal value of 0.95 for each method. Coverage probabilities within this range, denoted as the tolerable range below, were considered acceptable.

## Results

**FIGURE 5:**
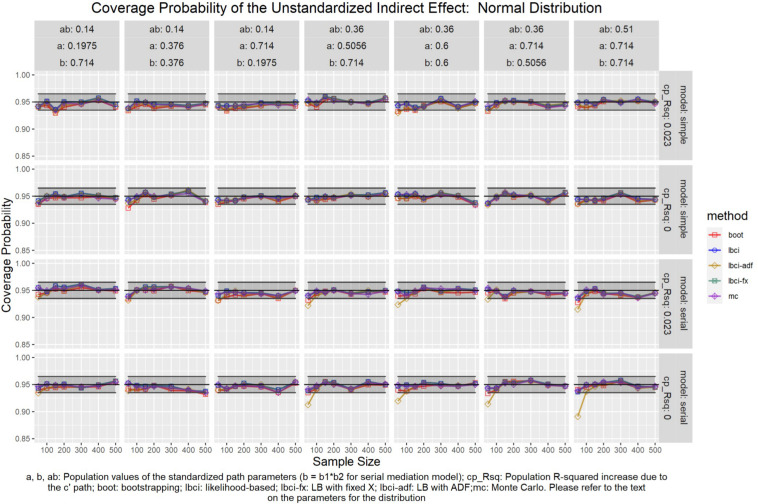
Normal Distribution (Unstandardized Indirect Effect). a, b, ab: Population values of the standardized path parameters (b = b1 × b2 for serial mediation model); cp_Rsq: Population R-squared increase due to the c′ path; boot: bootstrapping; Ibci: likelihood-based; Ibci-fx: LB with fixed X; Ibci-adf: LB with ADF;mc: Monte Carlo. Please refer to the text on the parameters for the distribution.

**FIGURE 6:**
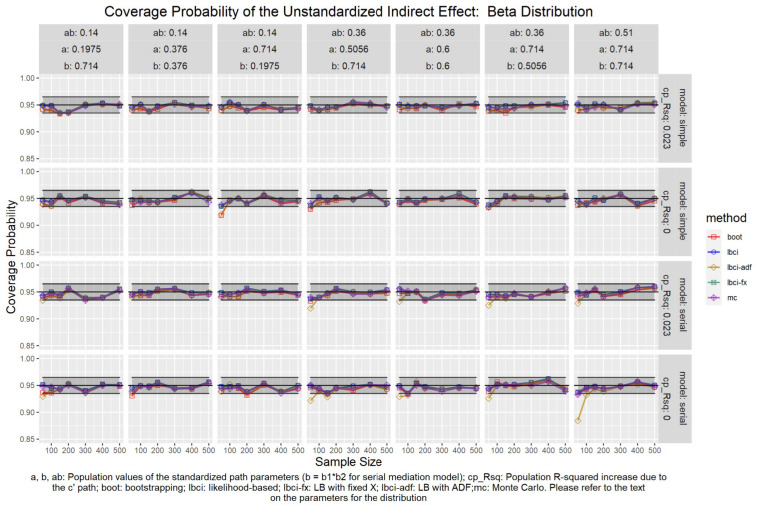
Beta Distribution (Unstandardized Indirect Effect). a, b, ab: Population values of the standardized path parameters (b = b1 × b2 for serial mediation model); cp_Rsq: Population R-squared increase due to the c′ path; boot: bootstrapping; Ibci: likelihood-based; Ibci-fx: LB with fixed X; Ibci-adf: LB with ADF;mc: Monte Carlo. Please refer to the text on the parameters for the distribution.

**FIGURE 7:**
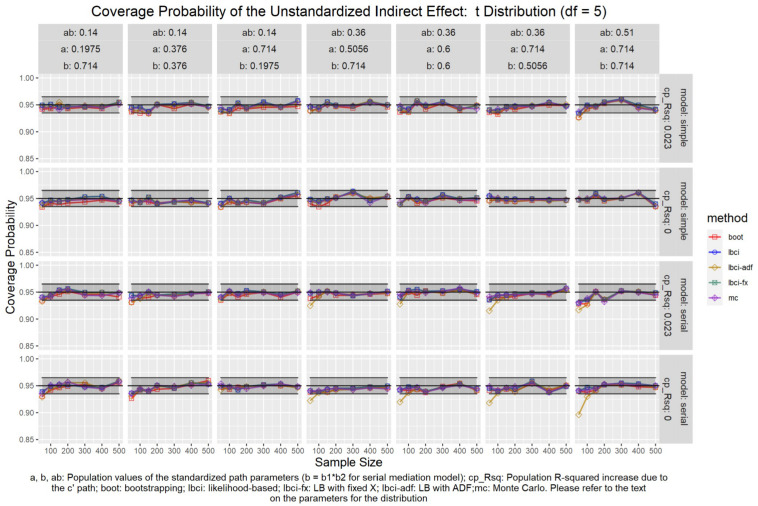
*t*(5) Distribution (Unstandardized Indirect Effect). a, b, ab: Population values of the standardized path parameters (b = b1 × b2 for serial mediation model); cp_Rsq: Population R-squared increase due to the c′ path; boot: bootstrapping; Ibci: likelihood-based; Ibci-fx: LB with fixed X; Ibci-adf: LB with ADF;mc: Monte Carlo. Please refer to the text on the parameters for the distribution.

**FIGURE 8:**
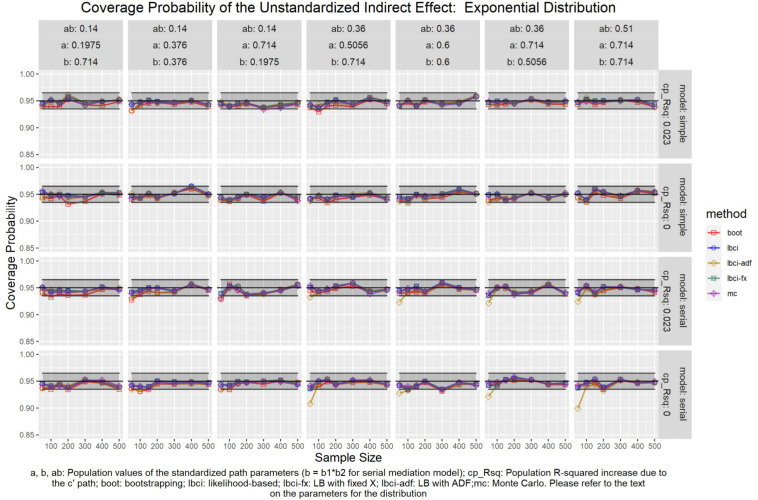
Exponential Distribution (Unstandardized Indirect Effect). a, b, ab: Population values of the standardized path parameters (b = b1 × b2 for serial mediation model); cp_Rsq: Population R-squared increase due to the c′ path; boot: bootstrapping; Ibci: likelihood-based; Ibci-fx: LB with fixed X; Ibci-adf: LB with ADF;mc: Monte Carlo. Please refer to the text on the parameters for the distribution.

**FIGURE 9:**
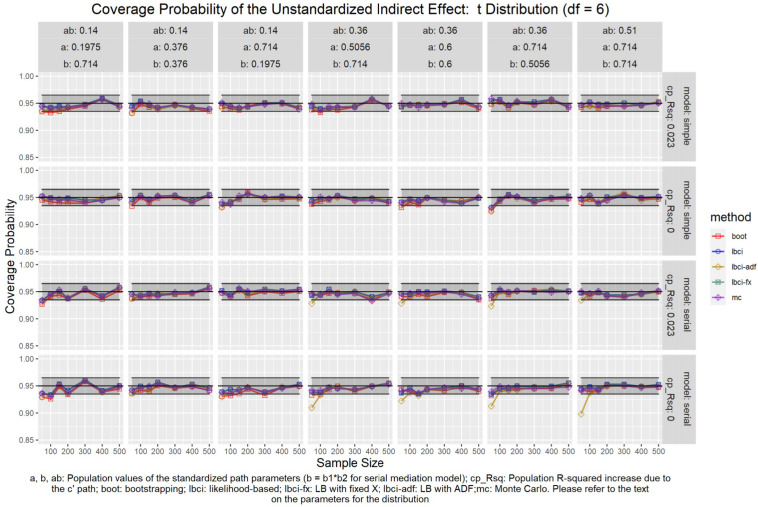
*t*(6) Distribution (Unstandardized Indirect Effect). a, b, ab: Population values of the standardized path parameters (b = b1 × b2 for serial mediation model); cp_Rsq: Population R-squared increase due to the c′ path; boot: bootstrapping; Ibci: likelihood-based; Ibci-fx: LB with fixed X; Ibci-adf: LB with ADF;mc: Monte Carlo. Please refer to the text on the parameters for the distribution.

**FIGURE 10:**
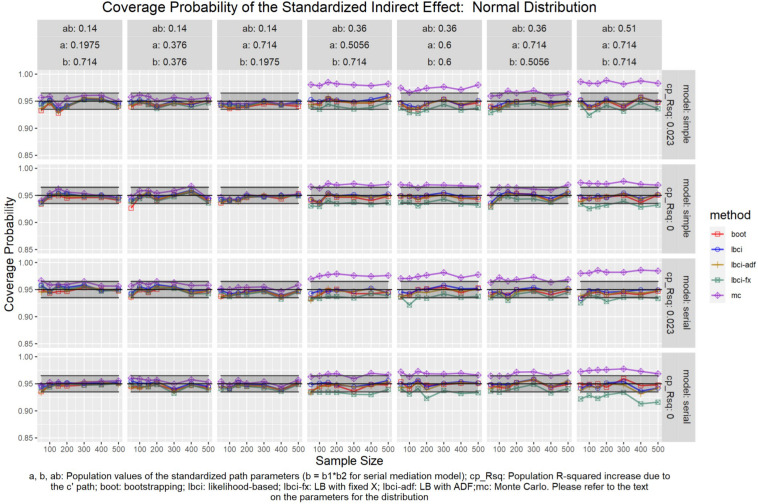
Normal Distribution (Standardized Indirect Effect). a, b, ab: Population values of the standardized path parameters (b = b1 × b2 for serial mediation model); cp_Rsq: Population R-squared increase due to the c′ path; boot: bootstrapping; Ibci: likelihood-based; Ibci-fx: LB with fixed X; Ibci-adf: LB with ADF;mc: Monte Carlo. Please refer to the text on the parameters for the distribution.

**FIGURE 11:**
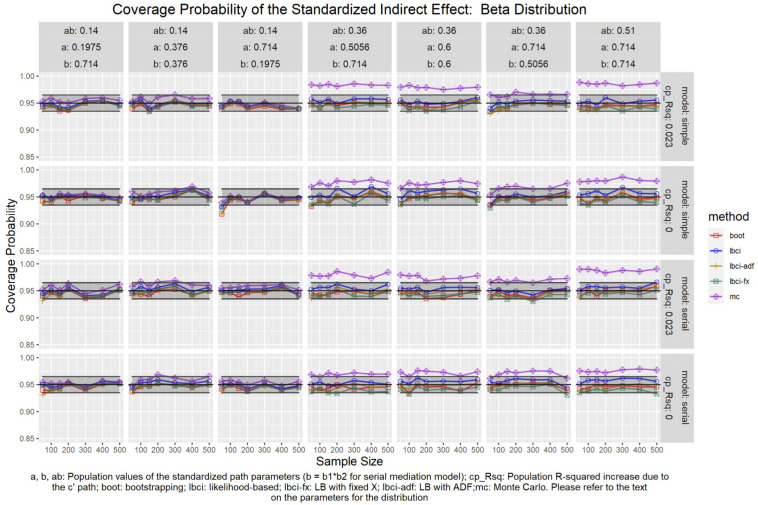
Beta Distribution (Standardized Indirect Effect). a, b, ab: Population values of the standardized path parameters (b = b1 × b2 for serial mediation model); cp_Rsq: Population R-squared increase due to the c′ path; boot: bootstrapping; Ibci: likelihood-based; Ibci-fx: LB with fixed X; Ibci-adf: LB with ADF;mc: Monte Carlo. Please refer to the text on the parameters for the distribution.

**FIGURE 12:**
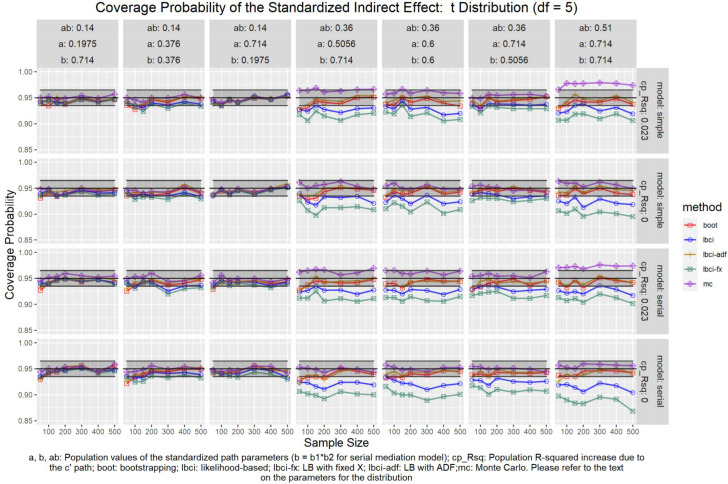
*t*(5) Distribution (Standardized Indirect Effect). a, b, ab: Population values of the standardized path parameters (b = b1 × b2 for serial mediation model); cp_Rsq: Population R-squared increase due to the c′ path; boot: bootstrapping; Ibci: likelihood-based; Ibci-fx: LB with fixed X; Ibci-adf: LB with ADF;mc: Monte Carlo. Please refer to the text on the parameters for the distribution.

**FIGURE 13:**
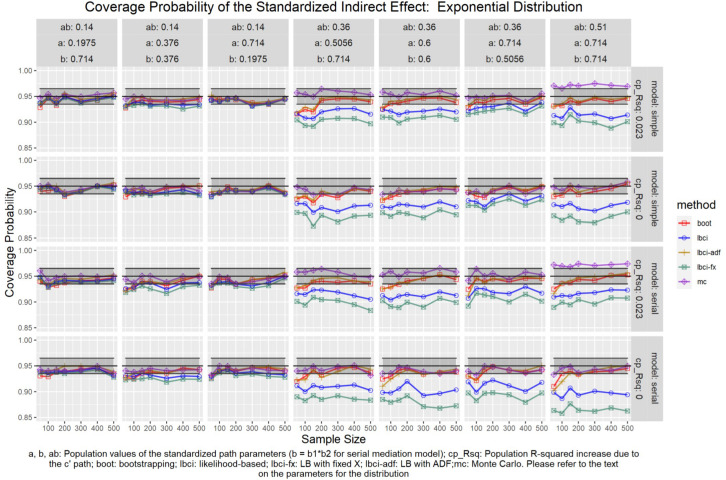
Exponential Distribution (Standardized Indirect Effect). a, b, ab: Population values of the standardized path parameters (b = b1 × b2 for serial mediation model); cp_Rsq: Population R-squared increase due to the c′ path; boot: bootstrapping; Ibci: likelihood-based; Ibci-fx: LB with fixed X; Ibci-adf: LB with ADF;mc: Monte Carlo. Please refer to the text on the parameters for the distribution.

**FIGURE 14:**
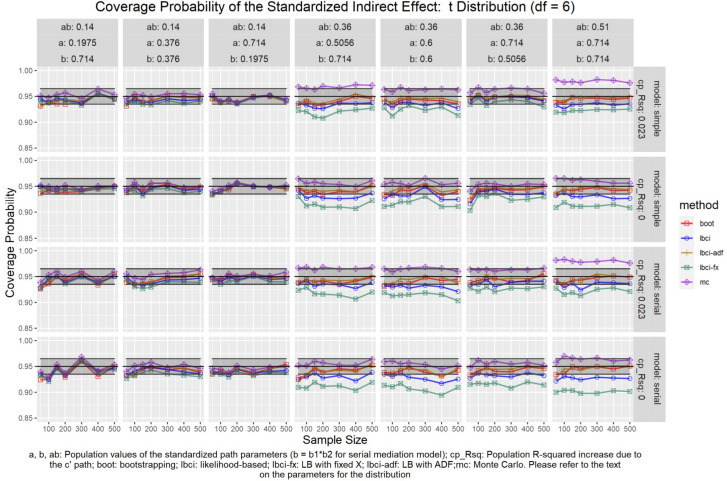
*t*(6) Distribution (Standardized Indirect Effect). a, b, ab: Population values of the standardized path parameters (b = b1 × b2 for serial mediation model); cp_Rsq: Population R-squared increase due to the c′ path; boot: bootstrapping; Ibci: likelihood-based; Ibci-fx: LB with fixed X; Ibci-adf: LB with ADF;mc: Monte Carlo. Please refer to the text on the parameters for the distribution.

The coverage probabilities for all five methods in all conditions were presented in Figures 5–14, except for two groups of conditions: conditions with both *a* and *b* paths zero in the population, and conditions with *R*^2^ increase due to the direct effect (*c*′) greater than 0.100. First, similar to previous studies (e.g., Cheung, 2009a), in these conditions, all methods have confidence intervals that were too wide and the coverage probabilities were close to one. Second, when the *R*^2^ increase due to direct effect was greater than 0.100, in which *ab* = 0.014, the patterns of results were similar to those with *R*^2^ increase equal to 0.023 or 0.000. Therefore, these conditions were not displayed. The graphs for all conditions can be found in the Supplementary Materials.

### Unstandardized Indirect Effect

As shown in Figures 5–9, all selected methods had coverage probabilities close to the nominal level (0.95), with the exceptions of LBCI-ADF, to be reported below. There were fluctuations in the coverage probabilities across conditions and a few conditions had coverage probabilities slightly below the tolerable range. However, no notable patterns were found and most of the miss rates were within the tolerable range. In short, even when the predictor was nonnormal, if the conditional distribution of the errors was normal, thus not violating the distributional assumptions in maximum likelihood or OLS, the common methods to form the confidence interval for the indirect effect still have optimal performance.

Unlike the other methods, LBCI-ADF had coverage probabilities lower than the optimal value in the serial mediation model when the standardized *b* path was large (0.7746 or 0.8450) and the sample size was 50, even when the predictor was normal distributed. The coverage probability could be lower than 90% when both the *a* path and *b* path were large and the sample size was 50. However, when the sample size was 100 or larger, the coverage probabilities of LBCI-ADF were within the tolerable range in the serial mediation model.

### Standardized Indirect Effect

#### Nonparametric Bootstrapping

For nonparametric bootstrapping, the coverage probabilities were within the tolerable range in both the simple mediation model and the serial mediation model when the predictor was normally distributed (Figure 10). This replicated the results in previous studies (e.g., Cheung, 2009a). When the predictor had a symmetric beta distribution with light tails, the nonparametric bootstrapping percentile confidence interval still had coverage probabilities within the tolerable range in both models (Figure 11). However, when the predictor was drawn from a *t*(5) distribution, which has heavy tails, the nonparametric bootstrapping percentile confidence interval tended to have coverage probabilities slightly below the tolerable range in both models (Figure 12), especially when the sample size was small (50 to 100). This tendency was more apparent when the predictor was drawn from an exponential distribution, which is positively skewed and has a heavy tail (Figure 13). In these conditions, if the standardized indirect effect was 0.36, the coverage probabilities could be as low as 0.925. When the predictor was drawn from a *t*(6) distribution, which has a smaller excess kurtosis (3) but is symmetric, the pattern was less obvious than that in the *t*(5) conditions (Figure 14). Despite the relatively worse performance compared to the conditions with normally distributed predictors, nonparametric bootstrapping percentile confidence interval generally had optimal to acceptable performance for all five distributions in both simple and serial mediation models when the sample size was 200 or higher.

#### LBCI

The LBCI for standardized indirect effects had coverage probabilities close to the nominal level for all conditions in both models when the predictor was normally distributed (Figure 10), replicating findings in previous studies (e.g., Cheung, 2009a). The lines for the LBCI and nonparametric bootstrapping substantially overlapped across conditions. Interestingly, in the serial mediation model with a normally distributed predictor, when the sample size was small (50 to 100), the coverage probabilities of the LBCI could even be closer to the nominal value than nonparametric bootstrapping did. When the predictor was drawn from a symmetric beta distribution with light tails (Figure 11), the LBCI tended to have coverage probabilities higher than those of nonparametric bootstrapping, though the coverage probabilities were still within the tolerable range in all conditions in both models. For the other two distributions with heavy tails [*t*(5) distribution and exponential distribution, Figures 12, 13], if the standardized indirect effect was small (0.14), the coverage probabilities of LBCI was still within the tolerable range in all conditions. However, when the standardized indirect effect was 0.36 or 0.51, the LBCI tended to be substantially too narrow, with coverage probabilities as low as 0.90 in some conditions. Moreover, the tendency to have low coverage probabilities did not lessen even if sample size increased, with coverage probabilities lower than 0.92 even with a sample size of 500 in some conditions. Last, when the predictor was drawn from a *t*(6) distribution, which has a smaller excess kurtosis (Figure 14), the coverage probabilities of LBCI were closer to the tolerable range in both models, but were still lower than the lower bound of the tolerance in some conditions, especially when the model is larger, with more variables (the serial mediation model).

#### LBCI-ADF

For both the simple mediation model and the serial mediation model, the LBCI-ADF method had coverage probabilities nearly indistinguishable from those of nonparametric bootstrapping in most conditions. When the differences were noticeable, usually it was the LBCI-ADF that had coverage probabilities closer to the nominal level than those of nonparametric bootstrapping. LBCI-ADF had coverage probabilities close to the nominal level even when the distribution was *t*(5) or exponential (Figures 12, 13), which both have excess kurtosis equal to 6, except when the sample sizes were small and the predictor was drawn from an exponential distribution. Nevertheless, in these conditions, the LBCI-ADF, similar to nonparametric bootstrapping, still had coverage probabilities closest to the nominal level than the other methods.

#### LBCI-Fixed-X

When the standardized indirect effect was small (0.14), the coverage probabilities of LBCI-Fixed-X were within or close to the tolerable range in most conditions, even when the predictor was drawn from an exponential distribution. When the standardized indirect effect was medium (0.36) or large (0.51), the coverage probabilities of LBCI-Fixed-X were still within the tolerable range when the predictor distribution was normal or beta (Figures 10, 11). However, when the predictor distribution was *t*(5) or exponential (Figures 12, 13), the coverage probabilities tended to be lower than the nominal value and exceed the tolerable range. The coverage probabilities could be as low as 0.90 in some conditions and could even be lower than 0.90 in the serial mediation model. It seems that sample size, even in the range of 100 to 500, did not have a strong and consistent impact on the coverage probabilities, suggesting that having a large sample could not help reduce this problem of suboptimal coverage of LBCI-Fixed-X for the two distributions with heavy tails.

#### Monte Carlo

The Monte Carlo confidence interval for the standardized indirect effect had miss rates consistently within the tolerable range only when the standardized indirect effect was small (0.14). When the standardized indirect effect was 0.36 or 0.51, the coverage probabilities could be far above the tolerable range (too liberal) even when the predictor was normally distributed (Figure 10). The same pattern was observed when the predictor was drawn from a symmetric beta distribution with light tails (Figure 11). Although the hit rate, unexpectedly, tended to move closer to the nominal level when the distribution of the predictor had heavy tails [*t*(5) distribution and exponential distribution, Figures 12, 13], we believe that this was merely due to the tendency to have a lower coverage probability due to heavy tailed distributions that offset the tendency of the Monte Carlo method to have a higher coverage probability.

## Discussion

The study demonstrated the performance of SEM-based LBCI methods to produce confidence intervals for unstandardized and standardized indirect effects under various kinds of nonnormal predictors in two mediation models. Results for unstandardized indirect effects showed that, if the nonnormality in variables were only due to nonnormality in predictors, as long as the assumption of normality of the conditional distributions of the errors are met, no notable departure from expected coverage probabilities was observed. If raw data are available, researchers can use either nonparametric bootstrapping or LBCI with maximum likelihood estimation to form the confidence interval of unstandardized indirect effect even if the predictor is nonnormal. If only the covariance matrix is available, then the Monte Carlo method or LBCI with maximum likelihood can be used.

However, results for standardized indirect effects showed some interesting patterns. First, LBCI with maximum likelihood performed well even in cases where the predictor was nonnormal when the effect size of the standardized indirect effect was small. Problems started to show, however, as in medium and large effect sizes especially in conditions where departure from normality was severe (e.g., *t* distribution and exponential distribution). Increasing the sample size did not seem to be helpful in estimating accurate confidence intervals in these situations. Therefore, although standardized indirect effect is just a simple transformation of unstandardized indirect effects, the LBCIs for them can perform substantially differently if the predictor is nonnormally distributed.

Second, LBCI-ADF performed the best across the LBCI methods, even slightly better than nonparametric bootstrapping in some conditions. Nonnormality did not have much influence on the estimation of confidence intervals for this procedure. This suggests that the LBCI method is still a viable option for standardized indirect effect with nonnormal predictor, although the suitable estimation method should be used. However, there are issues with using this method along with ADF distribution. First, when the departure from normality was extreme, cases where sample sizes were small produced lower hit rates when the effect size was medium or large. This is expected as the ADF estimator requires larger sample size compared to maximum likelihood due to the need to compute the fourth-order moments in the estimation. Second, the differences in results between the simple mediation model and the serial mediation model suggest that the sample size required for optimal performance of LBCI-ADF also depends on the number of variables. For a model with more variables, caution need to be taken in using the LBCI-ADF approach.

Third, LBCI-Fixed-X did not perform better than LBCI. In fact, in some cases, it performed much worse, most notably when the departure from normality was extreme. Therefore, unless the nonnormal predictor is genuinely a fixed variable in a study, fixing its variance can lead to worse performance. Given that fixing the exogenous variables may be the default in some programs, researchers may also unintentionally use the LBCI-Fixed-X for the standardized indirect effect, with the variance of the predictor fixed, resulting in suboptimal interval estimation when the predictor is nonnormal.

Last, Monte Carlo performed poorly for standardized indirect effect even when the predictor was normally distributed, except when the indirect effect was small. This finding is alarming because it means that, even for normally distributed predictor, this method can yield confidence interval that is too wide. Paradoxically, the larger the population effect, the wider the interval, and consequently the closer one of the confidence limits is to zero.

In sum, nonparametric bootstrapping and LBCI-ADF were the best-performing methods when the predictor was nonnormal, at least for the types of nonnormal investigated. Even in cases where the departure from normality for the predictor was extreme as in the case of the *t*(5) and exponential distributions, these methods still provided coverage probabilities within the tolerable range. Sample size, however, becomes relevant in the case of the exponential distribution, in as much as coverage probabilities were slightly below the tolerable range when sample sizes are small.

## Recommendations

Based on our findings, we recommend that, when multivariate nonnormality is present, researchers need to examine the nature and distribution of the variables to see whether the source of nonnormality is due to nonnormal errors of the mediator and/or outcome variable, or only due to the nonnormal predictor. This can be done with the usual techniques for assessing normality of residuals in regression models (e.g., Fox, 2016). If the normality is merely due to the nonnormal predictor, then all five methods yield confidence intervals for unstandardized indirect effect with coverage probabilities close to the nominal level across the conditions examined. Researchers can select methods suitable for their situations. If raw data are available, nonparametric bootstrapping can be used. If sample size is small (50 in the case of simple mediation model), then LBCI is preferred because it performed slightly better than nonparametric bootstrapping. If raw data are not available, then the Monte Carlo method can be used.

However, care must be taken if the confidence interval of standardized indirect effect is of interest, even if the only source of nonnormality is from the predictor. First, the Monte Carlo method, as implemented in the present study, should never be used. Second, all the other four methods still have optimal coverage probabilities when the distributions were nonormal but excess kurtosis was negative. For the distributions with excessive kurtosis, such as *t*(5) and *t*(6) distributions, which are symmetric, and exponential distribution, which is skewed, the LBCI and LBCI-Fixed-X can yield substantially low coverage probabilities when the standardized indirect effect was medium to high. Nonparametric bootstrapping and LBCI-ADF had similar performance and the coverage probabilities approached the nominal values as sample size increased. However, for the two distributions with high excess kurtosis, their coverage probabilities can be outside of the tolerable range even when the sample size was 150, which was large for a simple mediation model with just three variables. Therefore, even though nonparametric bootstrapping and ADF are distribution free in principle, they still need sufficient sample size to have optimal performance, and the results suggested that the larger the excess kurtosis, the larger the sample size requirement.

## Limitations

The present study had three limitations that deserve further investigation. First, we investigated only two models: a simple mediation model and a serial mediation model with two mediators. We followed the practice of some previous studies (e.g., Cheung, 2009a) to use the simple mediation model for investigation. This is a suitable starting point for studying the impact of nonnormal predictors. We also included a serial mediation model, which increased the number of variables while maintaining the comparability with the simple mediation in levels of indirect effect investigated. However, to enhance generalizability of the findings, further studies on more complicated models, such as parallel mediation models, need to be carried out to examine the impact of nonnormality and the minimal sample size required for optimal performance of distribution free methods, such as nonparametric bootstrapping and LBCI-ADF.

Second, we examined only three types of nonnormal distributions. We selected them because they have real-life counterparts and researchers are easier to see how these distributions can be related to their own situations. For example, variables such as depressive symptom scores in the general population (Tomitaka et al., 2017, 2018) and other similar clinical variables in psychology may follow a distribution that is highly skewed with a heavy tail. These variables can be approximated by the exponential distribution or similar skewed distributions. Variables with bounded scales are also common in psychology, such as variables measured as rates, percentages, or proportions. Likert-style rating scales are also common in psychological research and are used to measure a wide range of variables such as intelligence, personality, and psychological disorders. The sum or mean of these rating scales are likewise bounded. The double boundaries in the distribution of these variables may be better modeled using the beta distribution in the present study (Ferrari and Cribari-Neto, 2004). Last, sometimes the variables may be approximately symmetrically distributed in a study but there are some extreme values on both ends. This kind of distributions can be modeled by the *t* distribution, using the *df* to adjust the degree of extremeness on both ends. Although the distributions we selected covered distributions that are common in studies and qualitatively different, to understand better the impact of nonnormal predictors, other kinds of nonnormal distributions also need to be investigated.

Third, we did not include other robust methods recently developed for LBCI (e.g., Falk, 2018). They are not yet widely available in statistical packages or functions, but they may perform better than the distribution free methods we investigated. Further studies need to be conducted to examine the performance of these methods for nonnormal predictors in mediation models, especially for standardized indirect effects.

## Data Availability Statement

The raw data supporting the conclusion of this article will be made available by the authors, without undue reservation.

## Author Contributions

Both authors: writing and revising the manuscript. IP: contributed his expertise on mediation to the literature review and discussion. SF: conducted the simulation study and summarized the results.

## Conflict of Interest

The authors declare that the research was conducted in the absence of any commercial or financial relationships that could be construed as a potential conflict of interest.

## Footnotes

1 In some literature, *x* and *y* are called *independent variable* and *dependent variable*.

2 The kurtosis of the normal distribution is 3. Because it is common to use the normal distribution as the benchmark for comparison, we used excess kurtosis in this paper, which is kurtosis −3. The normal distribution has an excess kurtosis of 0.

3 https://osf.io/8gsw3/

## References

[B1] Baron R. M. Kenny D. A. (1986). The moderator-mediator variable distinction in social psychological research: conceptual, strategic, and statistical considerations. *J. Pers. Soc. Psychol.* 51 1173–1182. 10.1037/0022-3514.51.6.1173 3806354

[B2] Biesanz J. C. Falk C. F. Savalei V. (2010). Assessing mediational models: testing and interval estimation for indirect effects. *Multiv. Behav. Res.* 45 661–701. 10.1080/00273171.2010.498292 26735714

[B3] Blanca M. J. Arnau J. López-Montiel D. Bono R. Bendayan R. (2013). Skewness and kurtosis in real data samples. *Methodology* 9 78–84. 10.1027/1614-2241/a000057

[B4] Bollen K. A. (1989). *Structural Equations with Latent Variables.* Hoboken, NJ: John Wiley & Sons.

[B5] Bollen K. A. Stine R. (1990). Direct and indirect effects: classical and bootstrap estimates of variability. *Sociol. Methodol.* 20:115 10.2307/271084

[B6] Browne M. W. (1984). Asymptotically distribution-free methods for the analysis of covariance structures. *Br. J. Math. Statist. Psychol.* 37 62–83. 10.1111/j.2044-8317.1984.tb00789.x 6733054

[B7] Casella G. Berger R. (2001). *Statistical Inference*, 2nd Edn, Pacific Grove, CA: Cengage Learning, Inc.

[B8] Cheung M. W.-L. (2007). Comparison of approaches to constructing confidence intervals for mediating effects using structural equation models. *Struct. Equ. Model. Multidiscipl. J.* 14 227–246. 10.1080/10705510709336745

[B9] Cheung M. W.-L. (2009a). Comparison of methods for constructing confidence intervals of standardized indirect effects. *Behav. Res. Methods* 41 425–438. 10.3758/brm.41.2.425 19363183

[B10] Cheung M. W.-L. (2009b). Constructing approximate confidence intervals for parameters with structural equation models. *Struct. Equ. Model. Multidiscipl. J.* 16 267–294. 10.1080/10705510902751291

[B11] Cohen J. (1988). *Statistical Power Analysis for the Behavioral Sciences.* Hillsdale, NJ: Lawrence Erlbaum Associates.

[B12] Craig C. C. (1936). On the frequency function of xy. *Ann. Math. Statist.* 7 1–15. 10.1214/aoms/1177732541

[B13] Curran P. J. West S. G. Finch J. F. (1996). The robustness of test statistics to nonnormality and specification error in confirmatory factor analysis. *Psychol. Methods* 1 16–29. 10.1037/1082-989x.1.1.16

[B14] Efron B. Hastie T. (2017). *Computer Age Statistical Inference: Algorithms, Evidence, and Data Science.* Cambridge: Cambridge University Press.

[B15] Efron B. Tibshirani R. J. (1993). *An Introduction to the Bootstrap.* London: Chapman and Hall.

[B16] Falk C. F. (2018). Are robust standard errors the best approach for interval estimation with nonnormal data in structural equation modeling? *Struct. Equ. Model. Multidiscipl. J.* 25 244–266. 10.1080/10705511.2017.1367254

[B17] Falk C. F. Biesanz J. C. (2014). Inference and interval estimation methods for indirect effects with latent variable models. *Struct. Equ. Model. Multidiscipl. J.* 22 24–38. 10.1080/10705511.2014.935266 26736127

[B18] Ferrari S. L. P. Cribari-Neto F. (2004). Beta regression for modelling rates and proportions. *J. Appl. Statist.* 31 799–815. 10.1080/0266476042000214501

[B19] Fox J. (2016). *Applied Regression Analysis and Generalized Linear Models*, 3rd Edn, Thousand Oaks, CA: Sage.

[B20] Fritz M. S. Taylor A. B. MacKinnon D. P. (2012). Explanation of two anomalous results in statistical mediation analysis. *Multiv. Behav. Res.* 47 61–87. 10.1080/00273171.2012.640596 24049213 PMC3773882

[B21] Hayes A. F. (2017). *Introduction to Mediation, Moderation, and Conditional Process Analysis: A Regression-Based Approach*, 2nd Edn, New York, NY: Guilford Press.

[B22] Hayes A. F. Scharkow M. (2013). The relative trustworthiness of inferential tests of the indirect effect in statistical mediation analysis: does method really matter? *Psychol. Sci.* 24 1918–1927. 10.1177/0956797613480187 23955356

[B23] Hu L. Bentler P. M. Kano Y. (1992). Can test statistics in covariance structure analysis be trusted? *Psychol. Bull.* 112 351–362. 10.1037/0033-2909.112.2.351 1454899

[B24] Jöreskog K. G. (1973). “A general method for estimating a linear structural equation system,” in *Structural Equation Models in the Social Sciences*, eds Goldberger A. S. Duncan O. D. (Cham: Springer), 85–112.

[B25] MacKinnon D. P. (2008). *Multivariate Applications Series: Introduction to Statistical Mediation analysis.* Abingdon: Routledge.

[B26] MacKinnon D. P. Lockwood C. M. Hoffman J. M. West S. G. Sheets V. (2002). A comparison of methods to test mediation and other intervening variable effects. *Psychol. Methods* 7 83–104. 10.1037/1082-989x.7.1.83 11928892 PMC2819363

[B27] MacKinnon D. P. Lockwood C. M. Williams J. (2004). Confidence limits for the indirect effect: distribution of the product and resampling methods. *Multiv. Behav. Res.* 39 99–128. 10.1207/s15327906mbr3901_4 PMC2821115 20157642

[B28] Micceri T. (1989). The unicorn, the normal curve, and other improbable creatures. *Psychol. Bull.* 105 156–166. 10.1037/0033-2909.105.1.156

[B29] Neale M. C. Hunter M. D. Pritikin J. N. Zahery M. Brick T. R. Kirkpatrick R. M. (2015). OpenMx 2.0: extended structural equation and statistical modeling. *Psychometrika* 81 535–549. 10.1007/s11336-014-9435-8 25622929 PMC4516707

[B30] Neale M. C. Miller M. B. (1997). The use of likelihood-based confidence intervals in genetic models. *Behav. Genet.* 27 113–120. 10.1023/a:1025681223921 9145549

[B31] Pek J. Wu H. (2015). Profile likelihood-based confidence intervals and regions for structural equation models. *Psychometrika* 80 1123–1145. 10.1007/s11336-015-9461-1 25925009

[B32] Preacher K. J. Hayes A. F. (2004). SPSS and SAS procedures for estimating indirect effects in simple mediation models. *Behav. Res. Methods* 36 717–731. 10.3758/bf03206553 15641418

[B33] Preacher K. J. Kelley K. (2011). Effect size measures for mediation models: quantitative strategies for communicating indirect effects. *Psychol. Methods* 16 93–115. 10.1037/a0022658 21500915

[B34] Preacher K. J. Selig J. P. (2012). Advantages of Monte Carlo confidence intervals for indirect effects. *Commun. Methods Meas.* 6 77–98. 10.1080/19312458.2012.679848

[B35] R Core Team (2018). *R: A Language and Environment for Statistical Computing.* Vienna: R Foundation for Statistical Computing.

[B36] Rosseel Y. (2012). lavaan: an R package for structural equation modeling. *J. Statist. Softw.* 48 1–36. 10.1002/9781119579038.ch1

[B37] Serlin R. C. (2000). Testing for robustness in monte carlo studies. *Psychol. Methods* 5 230–240. 10.1037/1082-989x.5.2.230 10937332

[B38] Serlin R. C. Lapsley D. K. (1985). Rationality in psychological research: the good-enough principle. *Am. Psychol.* 40 73–83. 10.1037/0003-066x.40.1.73

[B39] Shrout P. E. Bolger N. (2002). Mediation in experimental and nonexperimental studies: new procedures and recommendations. *Psychol. Methods* 7 422–445. 10.1037/1082-989x.7.4.422 12530702

[B40] Sobel M. E. (1982). Asymptotic confidence intervals for indirect effects in structural equation models. *Sociol. Methodol.* 13 290–312. 10.2307/270723

[B41] Tomitaka S. Kawasaki Y. Ide K. Akutagawa M. Ono Y. Furukawa T. A. (2018). Distribution of item responses and total item scores for the Center for Epidemiologic Studies of depression Scale (CES-D): data from the irish longitudinal study on ageing (TILDA). *PLoS One* 13:e0202607. 10.1371/journal.pone.0202607 30114259 PMC6095586

[B42] Tomitaka S. Kawasaki Y. Ide K. Akutagawa M. Yamada H. Furukawa T. A. (2017). Exponential distribution of total depressive symptom scores in relation to exponential latent trait and item threshold distributions: a simulation study. *BMC Res. Notes* 10:614. 10.1186/s13104-017-2937-6 29169379 PMC5701434

[B43] Wu H. Neale M. C. (2012). Adjusted confidence intervals for a bounded parameter. *Behav. Genet.* 42 886–898. 10.1007/s10519-012-9560-z 22971875 PMC3486787

[B44] Yuan K.-H. Chan W. (2011). Biases and standard errors of standardized regression coefficients. *Psychometrika* 76 670–690. 10.1007/s11336-011-9224-6 27519686

